# Housing, sanitation and living conditions affecting SARS-CoV-2 prevention interventions in 54 African countries

**DOI:** 10.1017/S0950268821001734

**Published:** 2021-07-23

**Authors:** Timothy F. Brewer, Mary Zhang, David Gordon, Roger Yat-Nork Chung, Negussie Dejene, Cynthia L. Fonta, Tigist Grieve, Björn Halleröd, Richard Harris, Alba Lanau, Murray Leibbrandt, Yehualashet Mekonen, Bongai Muguni, Hector Najera, Shailen Nandy, S. Jody Heymann

**Affiliations:** 1 Department of Medicine, Geffen School of Medicine and Department of Epidemiology, Fielding School of Public Health, University of California, Los Angeles, Los Angeles, USA; 2 Bristol Poverty Institute, Townsend Centre for International Poverty Research, University of Bristol, Bristol, UK; 3 School of Public Health and Primary Care, The Chinese University of Hong Kong, Hong Kong, People's Republic of China; 4 African Child Policy Forum, Addis Ababa, Ethiopia; 5 Department of Sociology and Work Science, University of Gothenburg, Gothenburg, Sweden; 6 Centre d'Estudis Demogràfics, Barcelona, Spain; 7 African Centre of Excellence for Inequalities Research, University of Cape Town, Cape Town, South Africa; 8 Universidad Nacional Autónoma de México, Mexico City, Mexico; 9 School of Social Sciences, Cardiff University, Cardiff, UK; 10 Department of Health, Policy & Management, Fielding School of Public Health and Department of Public Policy, Luskin School of Public Affairs, University of California, Los Angeles, CA, USA

**Keywords:** COVID-19, non-pharmacologic interventions, prevention, Africa, public health

## Abstract

The feasibility of non-pharmacological public health interventions (NPIs) such as physical distancing or isolation at home to prevent severe acute respiratory syndrome coronavirus 2 (SARS-CoV-2) transmission in low-resource countries is unknown. Household survey data from 54 African countries were used to investigate the feasibility of SARS-CoV-2 NPIs in low-resource settings. Across the 54 countries, approximately 718 million people lived in households with ⩾6 individuals at home (median percentage of at-risk households 56% (95% confidence interval (CI), 51% to 60%)). Approximately 283 million people lived in households where ⩾3 people slept in a single room (median percentage of at-risk households 15% (95% CI, 13% to 19%)). An estimated 890 million Africans lack on-site water (71% (95% CI, 62% to 80%)), while 700 million people lacked in-home soap/washing facilities (56% (95% CI, 42% to 73%)). The median percentage of people without a refrigerator in the home was 79% (95% CI, 67% to 88%), while 45% (95% CI, 39% to 52%) shared toilet facilities with other households. Individuals in low-resource settings have substantial obstacles to implementing NPIs for mitigating SARS-CoV-2 transmission. These populations urgently need to be prioritised for coronavirus disease 2019 vaccination to prevent disease and to contain the global pandemic.

## Introduction

As of 6th July 2021, coronavirus disease 2019 (COVID-19), caused by severe acute respiratory syndrome coronavirus 2 (SARS-CoV-2), has resulted in approximately 183 million cases and approximately 4 million deaths in more than 200 countries, areas and territories [1], although total mortality due to COVID-19 may be as high as 7 million deaths [2]. The global COVID-19 case fatality ratio approximates that of the 1918 H1N1 Influenza pandemic and regional SARS-CoV-2 outbreaks have overwhelmed the healthcare capacity of high- as well as low- and middle-income countries [3–5].

SARS-CoV-2 spreads primarily by respiratory droplets generated by behaviours such as coughing, sneezing or talking, although airborne and fomite transmission also occur [6–8]. Until effective vaccines are universally available, non-pharmacological public health interventions (NPIs) are the principal means by which governments prevent SARS-CoV-2 transmission in their populations. In addition to isolation of those infected and contact tracing and quarantine for those exposed, the World Health Organization (WHO) recommends physical distancing, masking in public places and hand washing as important NPIs that countries should employ for COVID-19 prevention and control [8]. Laboratory-based and observational studies suggest that physical distancing and the wearing of face masks may reduce SARS-CoV-2 transmission by at least 80% [9, 10], and in vitro data demonstrate that alcohol-based hand rubs at WHO recommended concentrations reduce SARS-CoV-2 infectious titres by 3- to 6-fold [11]. These measures, together with shelter-in-place restrictions, have reduced SARS-CoV-2 transmission and cases in several countries [12]. However, failure to maintain these measures has resulted in resurgent COVID-19 cases [13, 14].

Persons living in poverty are more likely to suffer severe disease, require hospitalisation, incur economic hardships or die during pandemics, including COVID-19, even in high-income countries [15]. Given projected shortages of SARS-CoV-2 testing kits in many African countries [16] and delays until vaccines will be widely available, WHO-recommended NPIs are the main COVID-19 prevention tools available to countries facing a resurgent pandemic. Many African countries have experienced rapid growth in their urban populations living in poverty that may lack the resources to implement WHO-recommended NPIs except for mask wearing, if available [17, 18]. Moreover, WHO has documented a recent rapid rise in cases in Africa amidst the spread of the Delta variant [1].

Using data from representative household surveys in 54 African countries, we examined living conditions that may affect individuals' ability to shelter-in-place, employ physical distancing or have access to soap and water for handwashing.

## Methods

Nationally representative household demographic and socioeconomic survey or census data were used to create vulnerability indices regarding individuals' feasibility to implement COVID-19 NPIs. For each country, the most recent available national survey data with detailed living conditions information were used. Multiple Indicator Cluster Survey (MICS) or Demographic and Health Survey (DHS) data from 2010 to 2020 were available for 45 of 54 African countries (83%) [19, 20]. For countries with multiple surveys during this period, only the most recent survey was used. For Somalia data were drawn from the 2011 Somliland and Northeast Zone MICS4 surveys. Botswanan results were taken from the Botswana Multi-topic Household Survey of 2015–2016. MICS or DHS data were not available for Eritrea, Libya, Mauritius and Seychelles. Other representative household survey and census data from 2010 to 2020 were used to estimate living conditions in these countries. In 4 of 54 countries (7%), Cape Verde, Djibouti, Equatorial Guinea and Morocco, the most recent data were between 2000 and 2009 (Table 1).

**Table 1.** Household survey data by country, year, type and sample size

Country	Year	Survey	Unweighted sample size
Algeria	2019	MICS6^a^	151 745
Angola	2016	Standard DHS-VII^a^	73 914
Benin	2018	Standard DHS-VII	73 728
Botswana	2016	BMTHS^a^	24 119
Burkina Faso	2010	Standard DHS-VI	81 522
Burundi	2017	Standard DHS-VII	77 724
Cape Verde	2005	DHS (IDSR-II)	26 294
Cameroon	2018	Standard DHS-VII	57 624
Central African Republic	2019	MICS6	45 797
Chad	2019	MICS6	112 604
Comoros	2012	Standard DHS-VI	24 200
Congo	2014	MICS5	53 849
Côte d'Ivoire	2016	MICS5	56 522
Djibouti	2006	MICS3	28 014
Democratic Republic of the Congo	2017	MICS6	103 422
Egypt	2014	Standard DHS-VI	117 536
Equatorial Guinea	2000	MICS2	21 136
Eritrea	2010	EPHS^a^	133 387
Eswatini	2014	MICS5	21 024
Ethiopia	2016	Standard DHS-VII	73 901
Gabon	2012	Standard DHS-VI	40 654
Gambia	2019	Standard DHS-VII	54 240
Ghana	2018	MICS6	61 254
Guinea	2018	Standard DHS-VII	49 120
Guinea-Bissau	2019	MICS6	49 172
Kenya	2014	Standard DHS-VII	151 093
Lesotho	2018	MICS6	35 110
Liberia	2019	Standard DHS-VII	41 423
Libya	2018/2020	GWP & MSNA^a^	10 079
Madagascar	2018	MICS6	82 875
Malawi	2016	Standard DHS-VII	119 326
Mali	2018	Standard DHS-VII	54 115
Mauritania	2015	MICS5	67 156
Mauritius^b^	2017	HBS^a^	23 781
Morocco	2004	Standard DHS-IV	62 891
Mozambique	2011	Standard DHS-VI	61 842
Namibia	2013	Standard DHS-VI	40 548
Niger	2012	Standard DHS-VI	63 776
Nigeria	2018	Standard DHS-VII	186 450
Rwanda	2015	Standard DHS-VII	54 017
São Tomé and Príncipe	2019	MICS6	13 957
Senegal	2019	Continuous DHS-VIII	40 013
Seychelles^c^	2010	Census 2010	88 945
Sierra Leone	2019	Standard DHS-VII	71 645
Somalia^d^	2011	MICS4	59 381
South Africa	2016	Standard DHS-VII	37 925
South Sudan	2010	MICS4	55 973
Sudan	2014	MICS5	97 049
United Republic of Tanzania	2016	Standard DHS-VI	62 515
Togo	2017	MICS6	34 988
Tunisia	2018	MICS6	44 276
Uganda	2016	Standard DHS-VII	89 202
Zambia	2018	Standard DHS-VII	64 302
Zimbabwe	2019	MICS6	44 472
**Total**			**3 471 627**

Notes:.

a MICS = Multiple Indicator Cluster Survey; DHS = Demographic and Health Survey; BMTHS = Botswana Multi-topic Household Survey; EPHS = Eritrea Population and Health Survey; GWP = Gallup World Poll; MSNA = Multi-Sector Needs Assessment; HBS = Household Budget Survey.

b The estimates for Mauritius were based on the 2017 Household Budget Survey (HBS) and the 2011 Census data. However, the sample size presented in this table, which is 23 781, only includes the number of individuals in the HBS 2017. To avoid confusion, the number of individuals in the Census 2011 is not included in the survey population total.

c The estimates for Seychelles are from the 2010 Census except for overcrowding (⩾6 household members) which are taken from a sample of 1200 households in Q1 of the 2019 Labour Force Survey.

d Somalia MICS4 (2011) data contain the data from Somaliland and Northeast Zone.

### Variables

Variables were selected to assess the feasibility of physical distancing, routine handwashing, isolation or quarantine at home, as well as whether household conditions place multiple generations at risk. To assess the feasibility of physical distancing, we relied on measures of whether the household was shared by ⩾6 individuals and whether ⩾3 individuals shared a single sleeping room. The feasibility of regular handwashing was assessed by utilising measures of whether the household had piped, well or spring water within the dwelling or plot and whether the interviewer observed the presence of soap and washing facilities in the home. The feasibility of isolating or going into quarantine at home was based on whether the household had a refrigerator and/or cooking facilities and whether the household had toilet facilities in the home or plot not shared with other households. The risk of intergenerational transmission was assessed by whether people >60 years old lived in households with ⩾3 younger individuals.

### Data analyses

The percentage of at-risk people in each country was calculated, as was the distribution of at-risk people across all 54 African countries. Summary median at-risk percentages for all 54 countries and 95% confidence intervals were determined using the bias-corrected and accelerated bootstrap method. To estimate the potential total at-risk population per country, the survey data were re-weighted by five-year age groups and gender using the United Nations African country population estimates for 2020 [21]. To assess the effect of survey data on the results, sensitivity analyses were performed limiting the analyses to the 35 countries (65%) with data from 2015 or later.

Ethics approval was obtained by the institutions that administered the surveys according to their national requirements. All analyses used anonymised survey data.

## Results

Household survey data were available for all 54 African countries. The individual country sample sizes ranged from 10 079 (Libya)^1^ to 186 450 (Nigeria). In total, data were available for 3 471 627 individuals (Table 1).

### Physical distancing feasibility

In 36 of 54 African countries, 50% or more of the population lived in households with ⩾6 people at the time of the most recent household survey. In eight countries, ⩾70% of the population lived in households with six or more people. Across the 54 countries, the median number of households with ⩾6 people present was 56.4% (95% confidence interval (CI), 51.2% to 59.8%). At the individual country level, large households ranged from a low of 14.0% in Mauritius to a high of 86.1% in Senegal (see Supplemental Table 1 for additional details).

In 41 out of 51 African countries for which data were available, 10% or more of the population lived in households where ⩾3 people shared a single sleeping room. In 28 of those countries, 15% or more of the population lived in such households. The median number of people living in households with ⩾3 people in a single sleeping room was 15.4% (95% CI, 12.5% to 19.4%), ranging at the country level from 1.5% of the population in Mauritius to 85.7% of people in Eritrea. Across the continent, approximately 718.2 million people lived in households with ⩾6 individuals at home while 282.8 million people lived in households with ⩾3 persons shared a single sleeping room (see Supplemental Table 1 for additional details).

### Handwashing feasibility

In 39 out of 54 African countries, ⩾50% of the population did not have access to water in their dwelling/plot. However, in 27 countries, this figure rose to ⩾70%. The median number of Africans that lacked access to water within their dwelling/plot was 70.6% (95% CI, 61.5% to 79.9%), ranging from 0.6% of the population in Mauritius to 92.9% of the population in Central African Republic (see Supplemental Table 2 for additional details).

Forty-three country surveys had data on whether soap and/or washing facilities were observed in the home. In 25 countries, ⩾50% of households were observed not to have soap or washing facilities in the home. In 16 countries, this rose to ⩾70% of the population. Across countries with available data, the median number of people lacking observed soap/washing facilities in the household was 55.9% (95% CI, 42.3% to 73.4%), ranging at the country level from 2.5% of people in Libya to 93.8% of people in Liberia. Overall, 889.5 million people in Africa lacked access to water in their home/plots while 700 million persons were observed to lack soap/washing facilities in their homes (see Supplemental Table 2 for additional details).

### Isolation and quarantine feasibility

Across Africa, the median number of people per country without a refrigerator in the home was 78.7% (95% CI, 66.7% to 88.1%), ranging at the country level from 2.0% of people in Mauritius to 98.7% of people in Central African Republic. The median number of people that lacked or shared a toilet with other households was 45.1% (95% CI, 39.3% to 51.7%) and 60.6% (95% CI, 51.3% to 67.6%) needed to collect or buy firewood for cooking or had no at-home cooking facilities (see Supplemental Table 3 for additional details).

### Multigenerational families

**Fig. 1.**
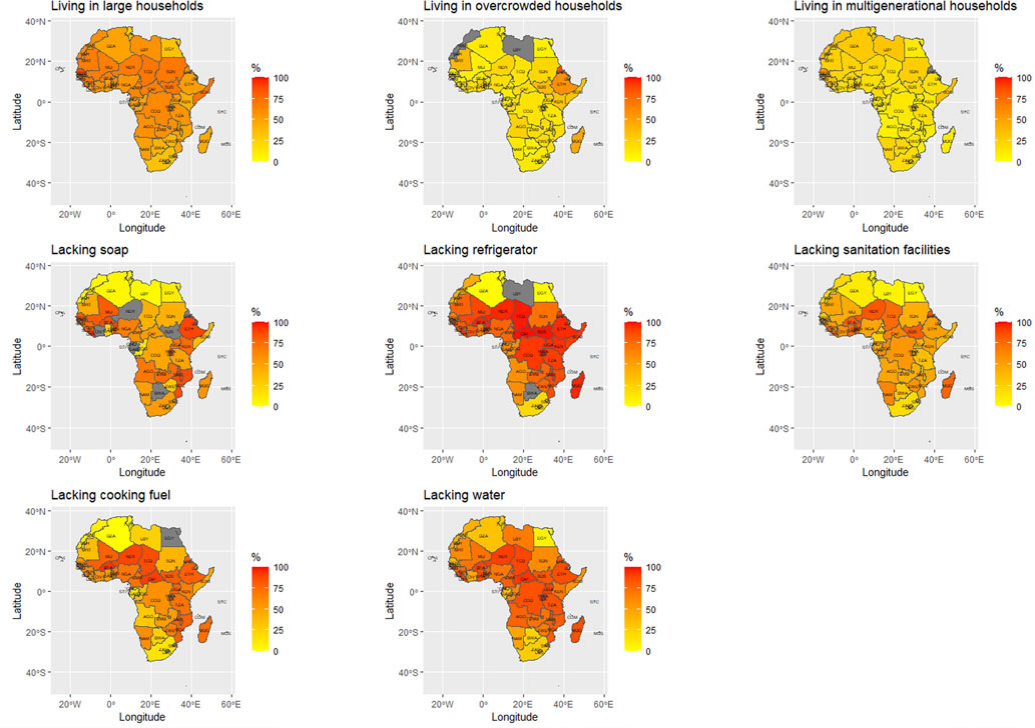
Proportion of population with resource challenges to implementing WHO recommended non-pharmaceutical interventions against COVID-19 by vulnerability indicator in 54 countries of Africa.

In 26 of the 51 countries with available data, 20% or more of the population lived in households including both persons >60 years old and ⩾3 younger individuals. The percentage of people in multigenerational households including persons >60 years old ranged from 8.5% in São Tomé and Príncipe to 55.8% in Senegal. Approximately 245.7 million people in Africa live in multigenerational households that include at least one person >60 years old. The percentages of people vulnerable to COVID-19 by indicator for each country are shown in Fig. 1.

### Sensitivity analyses

The analyses were repeated limiting the dataset to the 35 countries with results from 2015 or later. The point estimate for each variable outcome using this truncated dataset was the same or slightly higher than the results for the corresponding variables from the overall dataset (Table 2).

**Table 2.** Median percentage of African households with potential challenges to implementing non-pharmacologic interventions to prevent COVID-19

COVID-19 Metric	54 Countries		2015 or later surveys (35 countries)	
	Median	95% CI^a^	Median	95% CI^a^
⩾6 persons per household	0.56	0.51–0.60	0.56	0.50–0.59
⩾3 persons sharing a single sleeping room	0.15	0.13–0.19	0.15	0.12–0.19
Lacking water in dwelling/plot	0.71	0.62–0.80	0.75	0.68–0.84
No observed soap/washing facilities in household	0.56	0.42–0.73	0.62	0.47–0.74
No in-home refrigerator	0.79	0.67–0.88	0.82	0.74–0.90
No toilet in home	0.45	0.39–0.52	0.45	0.33–0.53
No in-home cooking facilities or need for firewood	0.61	0.51–0.68	0.63	0.52–0.70

a Confidence interval.

## Discussion

Until vaccines for COVID-19 are widely available in every country, NPIs are the primary tools for preventing SARS-CoV-2 transmission and associated COVID-19 morbidity and mortality. Implementing these measures entails ensuring that individuals are aware of COVID-19 prevention strategies including maintaining physical distancing of at least one metre away from members of other households, wearing face coverings, avoiding touching their faces or other people (such as by shaking hands) and washing their hands with soap/detergent as soon as they return home. However, these strategies require that people have sufficient space and resources to comply with these recommendations.

The results illustrate the substantial barriers many African households face in keeping safe from SARS-CoV-2 infection because of living conditions that preclude their ability to quarantine, isolate or maintain physical distancing and because of substantial obstacles to handwashing. When people need to leave their homes daily to access food, water, cooking or sanitation facilities, it is difficult (if not impossible) for them to isolate themselves in their homes for extended periods of time to avoid COVID-19 infections. Physical distancing policies – such as lockdowns – that depend on persons remaining in their homes for extended periods of time, are unlikely to be feasible or effective in many low-resource settings even when implemented only for short periods.

Approximately 718.2 million people (about 54% of the African population) lived in households with ⩾6 individuals at home, while 282.8 million people (~21% of the African population) slept in a room with ⩾3 persons. These COVID-19 vulnerability indicator results show that, in many African countries, large numbers of people are unlikely to be able to isolate within their homes, creating a high-risk for household transmission of SARS-CoV-2. Over 245.7 million individuals (about 18%) live in multigenerational households with at least one person >60 years old, placing millions of elderly Africans at risk for COVID-19 infection [22]. Over 889.5 million persons (about 66%) do not have in-home sources of water and 700 million persons (about 52%) lack soap and washing facilities. These conditions preclude routine handwashing, an essential non-pharmacological COVID-19 prevention measure. Moreover, additional risks for population-based COVID-19 spread exist for which data were not available for all countries. These risks include needing to access crowded markets for food and income and the necessary mobility of children, youth and adults outside of the household for education and work.

The use of nationally representative survey data for each country is a strength of this analysis. Though 35 of the 54 datasets used (65%) were based on surveys collected since 2015, four surveys were ⩾10 years old. Many sub-Saharan African countries have experienced substantial growth in their urban populations living in poverty during this period [17], and the older surveys may not reflect current population demographics. However, limiting the analyses to the 35 countries with survey data from 2015 or later gave results consistent with the overall dataset with any tendency towards change being a slight worsening of conditions.

Although the nationally representative surveys provide important data on living conditions, limitations of this study include the lack of information on working and transportation conditions. Additionally, risk for SARS-CoV-2 infection will be modulated by the relative amount of time susceptible persons spend indoors *vs.* outdoors when exposed to infectious persons. This modifying factor cannot be assessed using these data. The surveys do not include information regarding non-household and non-resident populations, which may have different risks for COVID-19 infections compared with household residents. The surveys also do not include information about home ventilation or the length of time household members spend in their homes, which may be independent SARS-CoV-2 risk factors. In some cases, population demographics and living conditions may have changed since the most recent nationally representative survey data were collected. However, limiting the analyses to the most recent surveys from 2015 or later suggests that living conditions are stable or getting worse.

As SARS-CoV-2 vaccine access expands in high-income countries, global inequality in access to vaccines is having devastating consequences as COVID-19 cases and deaths rise in middle- and low-income countries [1]. Given the magnitude of obstacles to implementing NPIs in countries across Africa, getting sufficient vaccine supplies to Africa urgently needs to be prioritised.

SARS-CoV-2 is the latest in a series of respiratory viral outbreaks to have caused substantial morbidity, mortality and economic disruption over the last two decades [23, 24]. Other recent respiratory virus outbreaks include the severe acute respiratory syndrome (SARS) and Middle East respiratory syndrome (MERS) coronaviruses and the H5N1 and H1N1 influenza viruses. Given the ongoing risk for respiratory viral epidemics and pandemics, long-term global resource commitments for combatting poverty, improving housing, water and sanitation, and scaling up vaccine production and ensuring access to vaccines for all people at high risk of infection, regardless of country of origin, are needed.

In conclusion, hundreds of millions of people across Africa lack means for implementing NPIs to prevent SARS-CoV-2 transmission. These findings raise the urgency of getting vaccines rapidly to all countries in Africa and for addressing the underlying conditions of poverty that place populations at increased risk for morbidity and mortality from respiratory virus outbreaks and pandemics.

## Data Availability

The study data are available from the Multiple Indicator Cluster Survey (MICS), Demographic and Health Survey (DHS), Botswana Multi-topic Household Survey (BMTHS), Eritrea Population and Health Survey (EPHS), Gallup World Poll 2015–2018 Libya microdata aggregated (GWP), Libya Multi-Sector Needs Assessment (MSNA), Mauritius Household Budget Survey (HBS) and Mauritius 2011 Census data. The availability of the data is upon registration and used under licences. Links for imputed data from open resources may be obtained by contacting MZ or DG.

## Footnotes

1 Libya's sample contains both Gallup World Poll (*N* = 4018) and Multi-Sector Needs Assessment survey data (*N* = 6061).

## Supplementary material

For supplementary material accompanying this paper visit http://dx.doi.org/10.1017/S0950268821001734. click here to view supplementary material
