# Supplementary material for: Housing, sanitation and living conditions affecting SARS-CoV-2 prevention interventions in 54 African countries
Source: Epidemiol Infect. 2021 Jul 23;149:e183. doi: 10.1017/S0950268821001734 (PMC8367861; doi:10.1017/S0950268821001734)
Supplement: Supplementary file 1 [file S0950268821001734sup001.docx]

Supplemental tables for *“Housing, Sanitation and Living Conditions Affecting SARS-CoV-2 Prevention Interventions in 54 African Countries”* (HYG-OM-11852-May-21)

Supplemental Table 1: Prevalence of living condition challenges to physical distancing, by country (%)

| **Country** | **Large households** | **Overcrowded households** |
| --- | --- | --- |
| Algeria | 49.2 | 12.3 |
| Angola | 58.5 | 25.0 |
| Benin | 60.6 | 15.4 |
| Botswana | 41.2 | 8.5 |
| Burkina Faso | 66.0 | 12.2 |
| Burundi | 55.3 | 10.6 |
| Cameroon | 62.9 | 11.6 |
| Cape Verde | 52.0 | -- |
| Central African Republic | 65.7 | 18.7 |
| Chad | 70.2 | 15.3 |
| Comoros | 64.4 | 11.9 |
| Congo | 47.9 | 21.7 |
| Congo (Democratic Republic) | 62.0 | 15.4 |
| Côte d'Ivoire | 56.9 | 18.5 |
| Djibouti | 66.9 | 25.7 |
| Egypt | 33.2 | 8.3 |
| Equatorial Guinea | 70.7 | 9.7 |
| Eritrea | 56.9 | 85.7 |
| Eswatini | 49.1 | 12.9 |
| Ethiopia | 51.2 | 58.1 |
| Gabon | 52.2 | 13.4 |
| Gambia | 84.3 | 3.4 |
| Ghana | 53.0 | 28.6 |
| Guinea | 71.7 | 10.5 |
| Guinea-Bissau | 77.0 | 6.4 |
| Kenya | 42.8 | 31.8 |
| Lesotho | 36.3 | 29.2 |
| Liberia | 53.3 | 19.5 |
| Libya | 57.4 | -- |
| Madagascar | 45.9 | 46.8 |
| Malawi | 46.3 | 22.8 |
| Mali | 66.4 | 9.1 |
| Mauritania | 67.5 | 37.9 |
| Mauritius | 14.0 | 1.5 |
| Morocco | 59.8 | -- |
| Mozambique | 48.4 | 18.3 |
| Namibia | 50.9 | 12.0 |
| Niger | 67.5 | 22.9 |
| Nigeria | 56.0 | 18.0 |
| Rwanda | 42.7 | 13.1 |
| São Tomé and Príncipe | 40.3 | 16.7 |
| Senegal | 86.1 | 5.4 |
| Seychelles | -- | 5.8 |
| Sierra Leone | 59.8 | 11.5 |
| Somalia | 71.6 | 32.3 |
| South Africa | 33.3 | 12.6 |
| South Sudan | 70.2 | 37.1 |
| Sudan | 69.8 | 21.8 |
| Togo | 49.9 | 20.4 |
| Tunisia | 24.9 | 9.6 |
| Uganda | 54.0 | 24.8 |
| United Republic of Tanzania | 56.4 | 14.1 |
| Zambia | 58.4 | 19.4 |
| Zimbabwe | 39.1 | 19.5 |

Supplemental Table 2: Prevalence of living condition challenges to handwashing, by country (%)

| **Country** | **Lacking soap/detergent** | **Lacking water** |
| --- | --- | --- |
| Algeria | 5.0 | 33.2 |
| Angola | 69.6 | 78.6 |
| Benin | 89.2 | 79.4 |
| Botswana | -- | 23.3 |
| Burkina Faso | 85.0 | 88.8 |
| Burundi | 92.7 | 92.6 |
| Cameroon | 58.8 | 79.9 |
| Cape Verde | -- | 53.4 |
| Central African Republic | 52.0 | 92.9 |
| Chad | 42.3 | 88.1 |
| Comoros | 77.3 | 43.1 |
| Congo | 14.6 | 63.3 |
| Congo (Democratic Republic) | 47.4 | 85.0 |
| Côte d'Ivoire | -- | 45.6 |
| Djibouti | -- | 21.0 |
| Egypt | 10.6 | 8.3 |
| Equatorial Guinea | -- | 88.2 |
| Eritrea | -- | 81.2 |
| Eswatini | 36.7 | 56.5 |
| Ethiopia | 88.9 | 86.3 |
| Gabon | -- | 36.9 |
| Gambia | 88.3 | 47.7 |
| Ghana | 22.2 | 85.3 |
| Guinea | 74.5 | 75.0 |
| Guinea-Bissau | 65.8 | 73.9 |
| Kenya | 74.8 | 69.2 |
| Lesotho | 20.2 | 67.8 |
| Liberia | 93.8 | 85.3 |
| Libya | 2.5 | 68.0 |
| Madagascar | 55.9 | 85.1 |
| Malawi | 87.9 | 87.6 |
| Mali | 81.5 | 71.6 |
| Mauritania | 44.7 | 61.5 |
| Mauritius | -- | 0.6 |
| Morocco | 4.3 | 42.7 |
| Mozambique | 85.9 | 80.8 |
| Namibia | 51.0 | 48.3 |
| Niger | -- | 90.6 |
| Nigeria | 66.8 | 69.6 |
| Rwanda | 91.8 | 90.0 |
| São Tomé and Príncipe | 23.3 | 65.5 |
| Senegal | 80.3 | 37.4 |
| Seychelles | -- | 4.4 |
| Sierra Leone | 74.4 | 87.9 |
| Somalia | 36.2 | 56.1 |
| South Africa | 52.7 | 27.6 |
| South Sudan | -- | 83.2 |
| Sudan | 42.2 | 60.8 |
| Togo | 35.6 | 79.9 |
| Tunisia | 3.5 | 29.5 |
| Uganda | 68.6 | 89.6 |
| United Republic of Tanzania | 49.1 | 84.4 |
| Zambia | 73.4 | 71.8 |
| Zimbabwe | 30.6 | 68.0 |

Supplemental Table 3: Prevalence of living condition challenges to isolation and quarantine, by country (%)

| **Country** | **Lacking refrigerator** | **No or sharing toilet** | **Lacking cooking fuel** |
| --- | --- | --- | --- |
| Algeria | 2.2% | 11.7 | 0.2 |
| Angola | 58.7 | 48.3 | 29.8 |
| Benin | 95.3 | 82.9 | 67.8 |
| Botswana | -- | 31.4 | 26.4 |
| Burkina Faso | 96.0 | 79.9 | 92.5 |
| Burundi | 98.1 | 17.4 | 77.6 |
| Cameroon | 76.5 | 27.3 | 72.5 |
| Cape Verde | 44.2 | 45.4 | 34.2 |
| Central African Republic | 98.7 | 65.2 | 90.5 |
| Chad | 97.7 | 74.8 | 86.3 |
| Comoros | 71.4 | 20.3 | 78.6 |
| Congo | 59.4 | 60.7 | 36.0 |
| Congo (Democratic Republic) | 92.0 | 54.7 | 58.7 |
| Côte d'Ivoire | 81.7 | 57.2 | 58.1 |
| Djibouti | 55.6 | 12.6 | 3.2 |
| Egypt | 2.7 | 2.3 | -- |
| Equatorial Guinea | -- | 80.1 | -- |
| Eritrea | 91.0 | 83.3 | 75.1 |
| Eswatini | 44.2 | 43.2 | 60.7 |
| Ethiopia | 95.0 | 50.1 | 79.6 |
| Gabon | 62.1 | 44.7 | 15.0 |
| Gambia | 52.5 | 26.2 | 65.9 |
| Ghana | 66.7 | 76.0 | 51.5 |
| Guinea | 81.8 | 54.5 | 60.6 |
| Guinea-Bissau | 86.8 | 44.5 | 67.1 |
| Kenya | 93.7 | 50.4 | 66.3 |
| Lesotho | 71.2 | 43.3 | -- |
| Liberia | 89.5 | 72.1 | 51.6 |
| Libya | -- | 6.0 | 25.0 |
| Madagascar | 95.8 | 77.3 | 73.7 |
| Malawi | 93.4 | 38.2 | 82.3 |
| Mali | 88.1 | 44.8 | 79.2 |
| Mauritania | 78.7 | 48.1 | 35.4 |
| Mauritius | 2.0 | 3.6 | 1.9 |
| Morocco | 45.0 | 22.5 | 9.1 |
| Mozambique | 86.3 | 47.7 | 78.9 |
| Namibia | 59.3 | 62.7 | 59.6 |
| Niger | 95.8 | 82.9 | 88.4 |
| Nigeria | 78.0 | 48.2 | 70.0 |
| Rwanda | 97.9 | 20.6 | 70.7 |
| São Tomé and Príncipe | 42.6 | 53.3 | 39.7 |
| Senegal | 65.7 | 30.4 | 53.9 |
| Seychelles | 6.4 | 1.8 | 1.5 |
| Sierra Leone | 86.7 | 74.5 | 67.4 |
| Somalia | 91.0 | 38.5 | 45.4 |
| South Africa | 20.9 | 18.6 | 15.1 |
| South Sudan | 98.3 | 80.4 | 81.0 |
| Sudan | 72.2 | 43.3 | 40.2 |
| Togo | 89.8 | 77.0 | 54.2 |
| Tunisia | 2.7 | 2.8 | 0.2 |
| Uganda | 94.4 | 39.3 | 77.3 |
| United Republic of Tanzania | 90.8 | 35.0 | 71.6 |
| Zambia | 77.0 | 42.6 | 51.2 |
| Zimbabwe | 77.4 | 57.1 | 67.6 |
